# Supplementary material for: Phospholipase D1 Attenuation Therapeutics Promotes Resilience against Synaptotoxicity in 12-Month-Old 3xTg-AD Mouse Model of Progressive Neurodegeneration
Source: Int J Mol Sci. 2023 Feb 8;24(4):3372. doi: 10.3390/ijms24043372 (PMC9967100; doi:10.3390/ijms24043372)
Supplement: Supplementary file 1 [file ijms-24-03372-s001.zip › ijms-2172983-supplementary.pdf]

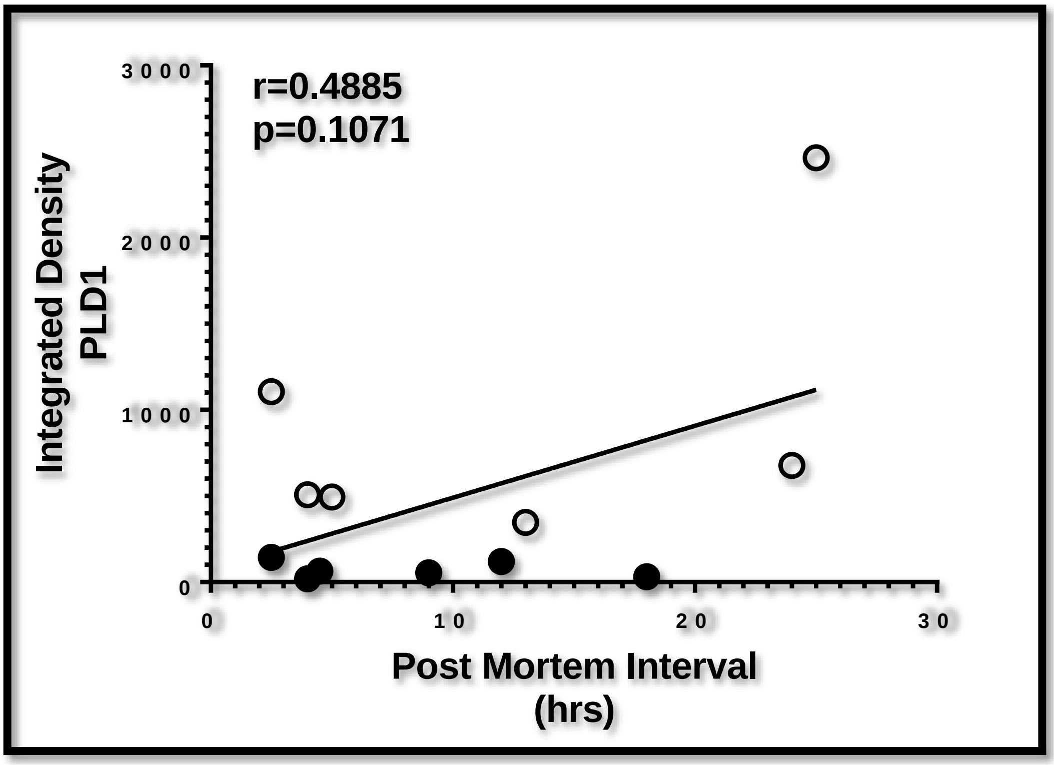

Suppl. Figure S1: The increase in PLD1 expression did not show a correlation with the postmortem interval. Correlation analysis between integrated density of PLD1 averaged for both Aβ and tau co-staining in the six control samples (filled circles) and six AD samples (clear circles) and postmortem interval values showed no significance (p=0.1071, Pearson's correlation test). Correlation coefficient (r=0.4885) shows no significant correlation of PLD1 expression with PMI, suggesting observed differences could not be attributed to differences in nonspecific postmortem tissue degradation.

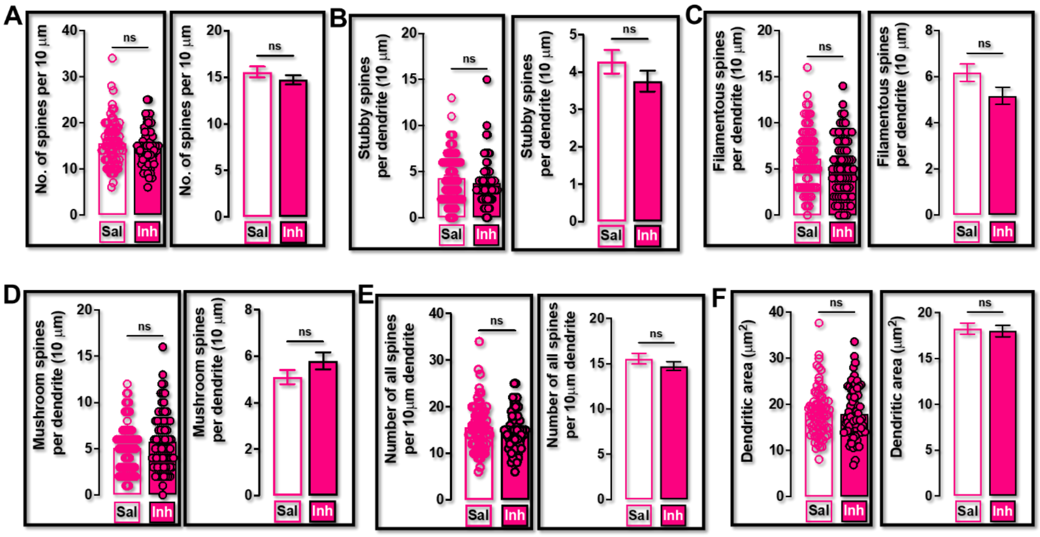

Suppl. Figure S2: Chronic PLD1 inhibition in 12-month-old 3xTg-AD mouse model of AD-like cognitive decline did not affect certain dendritic

parameters. A) Density of spines per 10 µm did not show any significant differences between the two treatment groups (p=0.3593). B) Number of stubby spines per 10 µm did not show any significant differences between the two treatment groups (p=0.2762). C) Number of filamentous spines per 10 µm did not show any significant differences between the two treatment groups (p=0.0663). D) Number of mushroom spines per 10 µm did not show any significant differences between the two treatment groups (p=0.0663). E) Number of all spines per 10 µm did not show any significant differences between the two treatment groups (p=0.3784). F) Dendritic area (µm^2) did not show any significant differences between the two treatment groups (p=0.7607). Each circle represents an individual spine. Saline circles have colored borders while inhibitor circles have black outline filled with color. For each animal, there were three slices assessed and for each slice there were five representative dendrites that were measured. Each panel has two graphs showing the same comparisons, the left shows the individual values in circles while the right shows the bar graph with the error bars. Values are expressed as Mean ± SEM. n=5 subjects per group (all females, 3 slices per animal and 5 representative dendrites per slice). Unpaired non-parametric Mann-Whitney test.

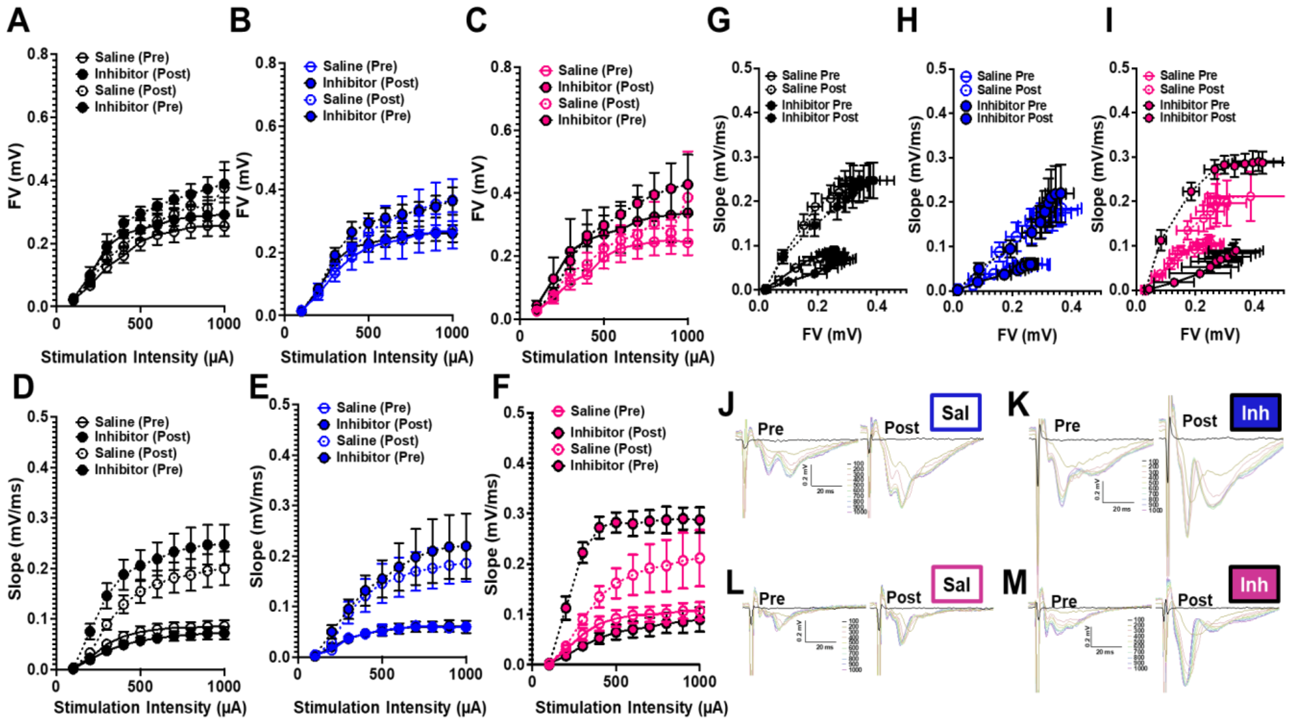

Suppl. Figure S3: Synaptic strength is not affected by the chronic regimen used for PLD1 inhibition studies in the 12-month-old 3xTg-AD mice at the Schaffer collateral synapse following HFS-LTP. Saline circles have colored borders while inhibitor circles have black outline filled with color. Pink color represents female, while blue represent male mice. The input – fiber volley (FV in millivolts (mV), panels A, B, C) and the output – slope (measured in mV/milliseconds, panels D, E, F) were done for each animal and plotted against the stimulation intensity (µA) on the X-axis. There was no statistically significant difference that was observed (see Suppl. Table 1 for values) between the treatment groups and before the HFS-LTP stimulation (pre) vs after (post) for A) all animals, or when the data was separated into B) males and C) females. As anticipated, there was a difference between the slopes before (pre, solid lines) and after (post, dotted lines) the HFS stimulation, however these differences were not significant between the treatments for D) all animals, or when the data was separated into E) males and F) females. Plotting the data in the input-output format where the slope was plotted as a function of the fiber volley at different stimulation intensity, there was a strong biological trend, as

seen with the slope, but no statistical difference before (pre, solid lines) and after (post, dotted lines) HFS in G) all animals or when separated into males (H) and females (I). Representative traces showing the input-output responses for J) saline treated male, K) inhibitor treated male, L) saline treated female and M) inhibitor treated female mice. Each panel provide two sets of traces with pre-HFS and post-HFS profiles (labeled Pre and Post respectively) for ten stimulations from 100-1000 µA (represented in an overlapping fashion in different colors). Scale bars showing the amplitude in mV (0.2mV) along the Y-axis and time in milliseconds (20ms) along the X-axis are provided for each panel. Values are expressed as Mean ± SEM, one-way ANOVA (Kruskal-Wallis test).

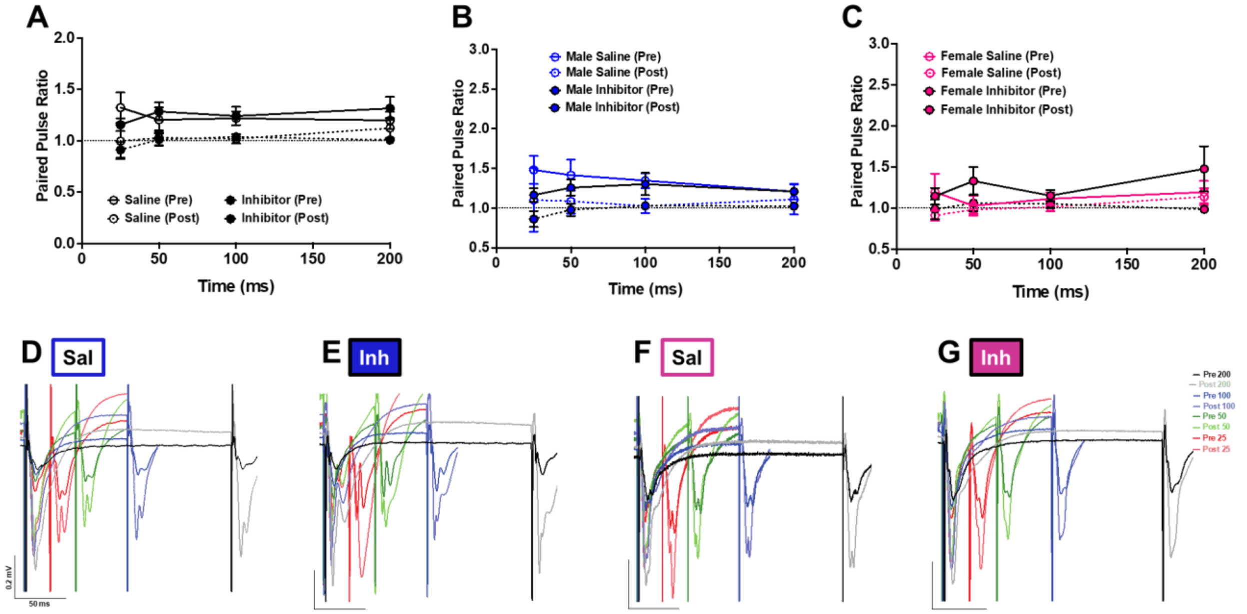

Suppl. Figure S4: Paired pulse facilitation does not show differences between the treatment groups. The slices were subjected to pre- and post-HFS assessment of presynaptic vesicle release by estimating whether there is a change in the slope between the first pulse and the second pulse when the time interval between the pulses is decreased from 200 ms to 100 ms to 50 ms to 25 ms (depicted along the Y-axis). A ratio greater than 1 indicates presynaptic changes. This paired pulse ratio (PPR) did not change between the treatment groups (saline in clear and inhibitor in solid) when considered either at pre (solid line) or post-HFS (dotted line) for A) all animals or when the data was separated into B) males and C) females. Representative traces showing the paired pulse facilitation for D) saline treated male, E) inhibitor treated male, F) saline treated female and G) inhibitor treated female mice. Each panel provide two overlapping sets of traces with pre-HFS and post-HFS profiles (represented in an overlapping fashion in different colors). Scale bars showing the amplitude in mV (0.2mV) along the Y-axis and time in milliseconds (50ms) along the X-axis are provided for each panel. Values are expressed as Mean ± SEM, one-way ANOVA (Kruskal-Wallis test).

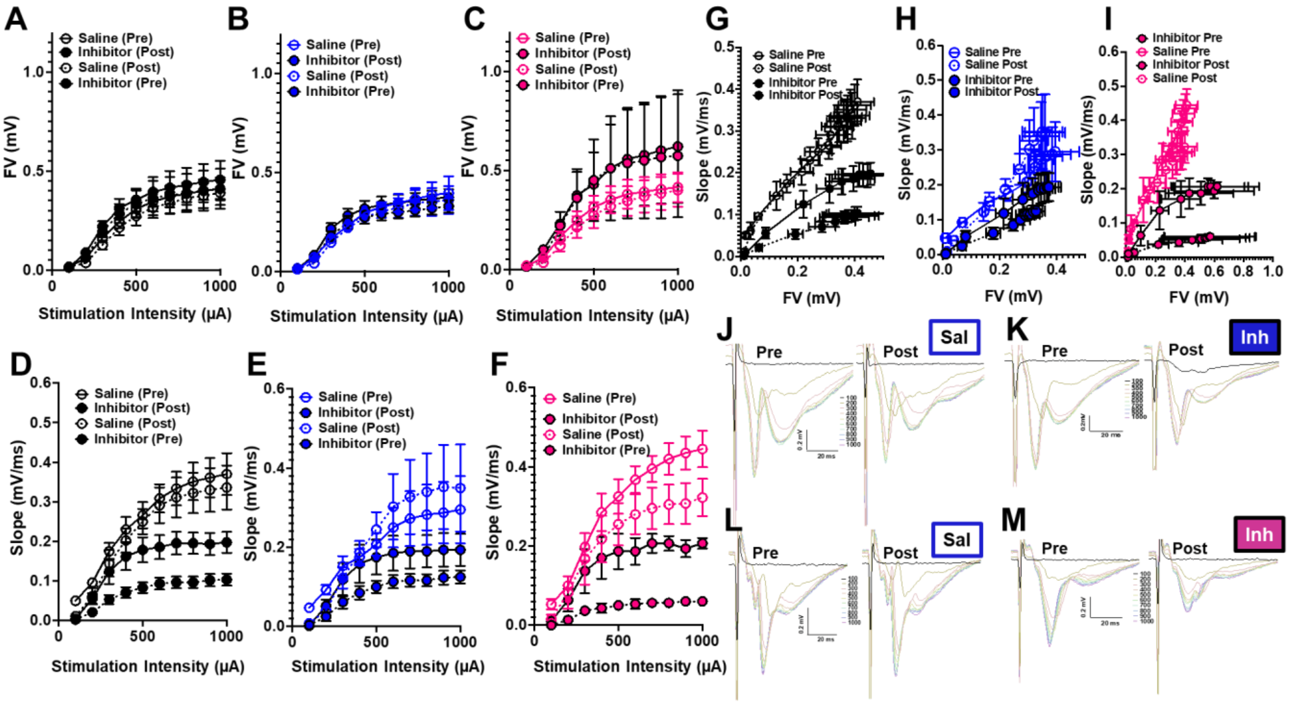

Suppl. Figure S5: Synaptic strength is not affected by the chronic regimen used for PLD1 inhibition studies in the 12-month-old 3xTg-AD mice at the Schaffer collateral synapse following LFS-LTD. The input – fiber volley (FV in millivolts (mV), panels A, B, C) and the output – slope (measured in mV/milliseconds, panels D, E, F) were done for each animal and plotted against the stimulation intensity (microamps) on the X-axis. There was no statistically significant difference that was observed between the treatment groups and before the HFS-LTP stimulation (pre, solid lines) vs after (post, dotted lines) for A) all animals, or when the data was separated into B) males and C) females. As anticipated, there was a difference between the slopes before (pre, solid lines) and after (post, dotted lines) the LFS stimulation, however these differences were not significant between the treatments (Suppl. Table 3) for D) all animals, or when the data was separated into E) males and F) females. Plotting the data in the input-output format where the slope was plotted as a function of the fiber volley at different stimulation intensity, there was a strong biological trend as seen with the slope but no statistical difference before (pre, solid lines) and after (post, dotted lines) LFS in G) all animals or when separated into males (H) and females (I). Representative traces showing the input-output responses for J) saline treated male, K) inhibitor treated male, L) saline treated female and M) inhibitor treated female mice. Each panel provide two sets of traces with pre-LFS and post-LFS profiles (labeled Pre and Post respectively) for ten stimulations from 100-1000 µA (represented in an overlapping fashion in different colors). Scale bars showing the amplitude in mV (0.2mV) along the Y-axis and time in milliseconds (20ms) along the X-axis are provided for each panel. Saline circles have colored borders while inhibitor circles have black outline filled with color. Pink color represents female, while blue represent male mice. Values are expressed as Mean ± SEM, one-way ANOVA (Kruskal-Wallis test).

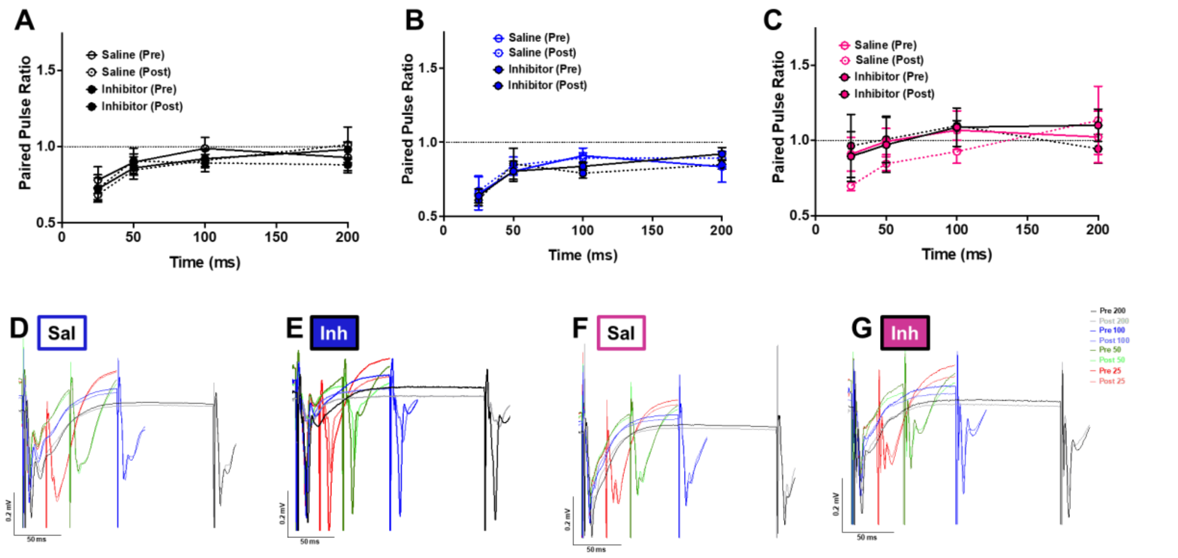

Suppl. Figure S6: Paired pulse depression does not show differences between the treatment groups. The slices were subjected to pre- and post-LFS assessment of presynaptic vesicle release by estimating whether there is a change in the slope between the first pulse and the second pulse when the time interval between the pulses is decreased from 200 ms to 100 ms to 50 ms to 25 ms (depicted along the Y-axis). The paired pulse ratio (PPR) did not change between the treatment groups (saline in clear and inhibitor in solid) when considered either at pre (solid line) or post-LFS (dotted line) for A) all animals or when the data was separated into B) males and C) females. Representative traces showing the paired pulse depression for D) saline treated male, E) inhibitor treated male, F) saline treated female and G) inhibitor treated female mice. Each panel provide two overlapping sets of traces with pre-LFS and post-LFS profiles (represented in an overlapping fashion in different colors). Scale bars showing the amplitude in mV (0.2mV) along the Y-axis and time in milliseconds (50ms) along the X-axis are provided for each panel. Values are expressed as Mean ± SEM, one-way ANOVA (Kruskal-Wallis test).

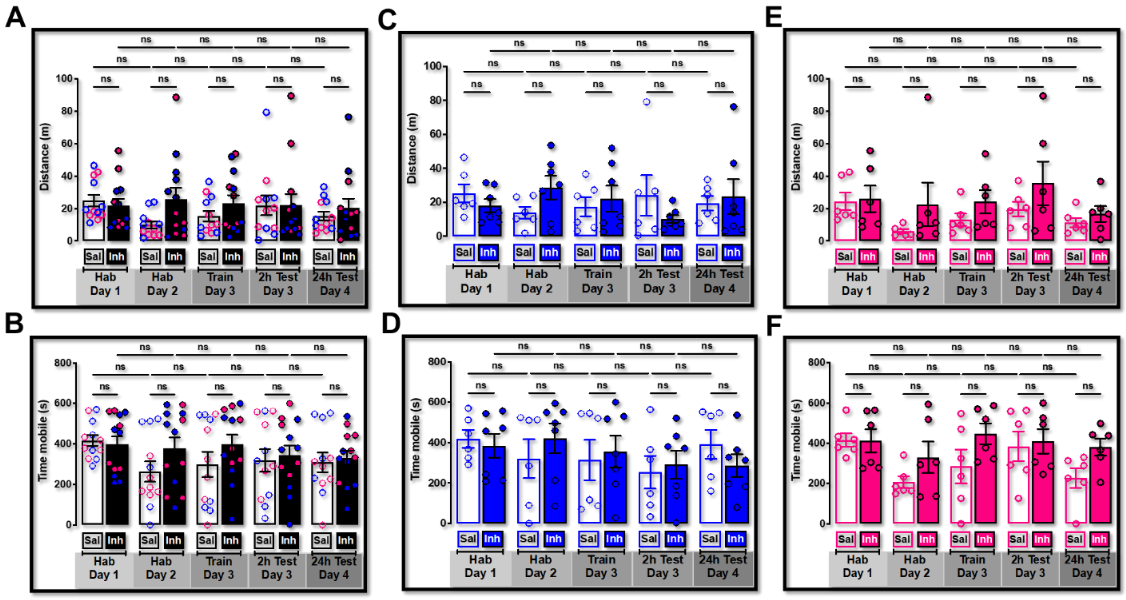

Suppl. Figure S7: PLD1 inhibitor associated NOR memory preservation in 12-month-old 3xTg-AD mice shows normal mobility and locomotor activity. There were no differences on any of the trials in A) the distance travelled, B) time mobile or time immobile (data not shown) between the groups, providing evidence for the absence of any non-specific effects confounding the

experimental measures. Separating the data into males (C, D) and females (E, F) did not show any differences in the distance travelled (C, E) or time mobile (D, F). Saline treated animals are shown in clear bars while inhibitor treated animals are shown in filled bars. Each circle represents a single animal. Saline circles have colored borders while inhibitor circles have black outline filled with color. Pink color represents female, while blue represent male mice. Values are expressed as Mean ± SEM, one-way ANOVA (Kruskal-Wallis test).

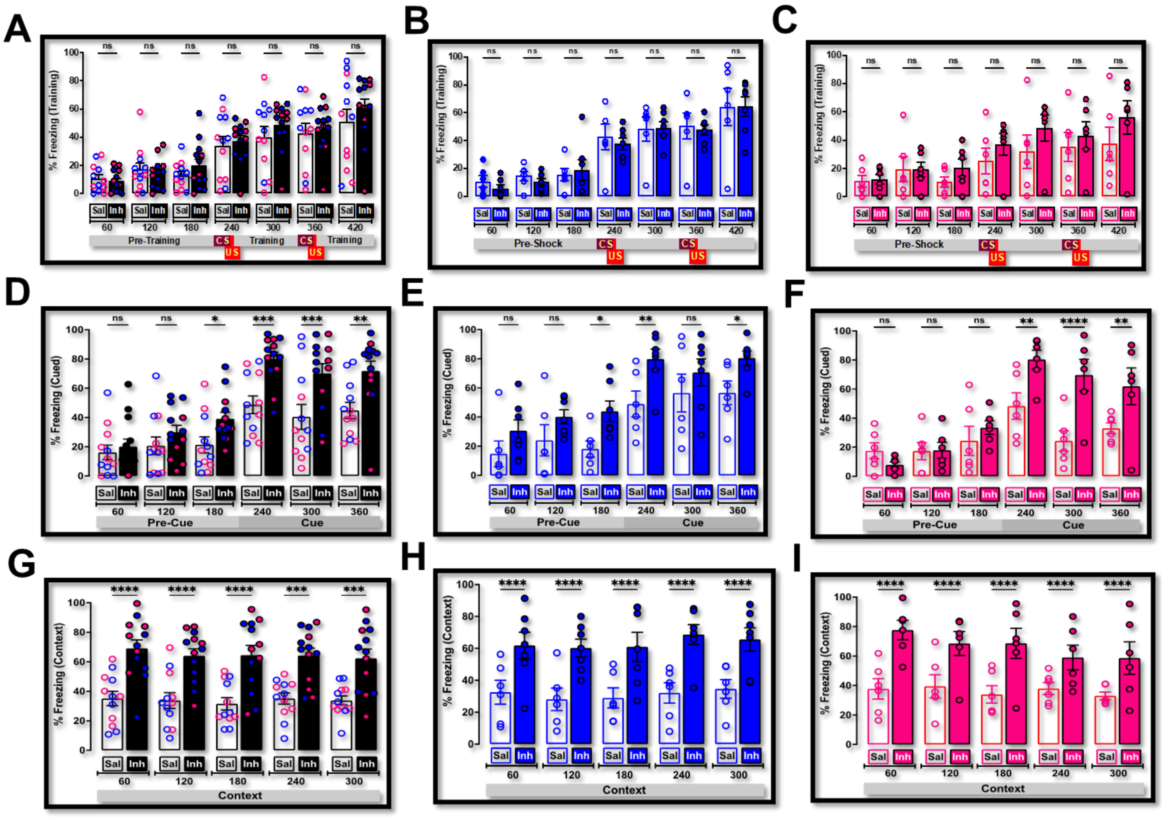

Suppl. Figure S8: Chronic PLD1 inhibition rescue of cued and contextual memory deficits in both male and female 12-month-old 3xTg-AD mice shows no differences in the pre-cue or training suggesting that VU01 does not have non-specific effects in the measured criteria. Percent freezing during training is shown in panel A) for all animals, B) for males and C) for females. There is no significant change between the two treatments within each epoch (shown in 60 seconds intervals on the X-axis), suggesting that there is no deficit or difference in the learning phase between the two treatment groups. Percent cued memory response (amygdala-dependent) does not show any differences between the treated groups in the pre-cue epochs (60, 120 and 180 seconds) in panel D) for all animals, E) for males and F) for females. However, the cue response shows a significant difference (240, 300 and 360 seconds) between the two treatment groups for D) all animals and F) females and in E) the male mice, except for 300 second epoch where the high variability in the freezing response of the saline group (clear or clear blue circles) shows greater distribution around the mean value. G) The contextual memory response (hippocampal based) is similar to the NOR response in which the difference between the treatment groups is significant at all five epochs all animals even when separated into H) males and I) females. Saline treated animals are shown in clear bars while inhibitor treated animals are shown in filled bars. Each circle represents a single animal. Saline circles have colored borders while inhibitor circles have black outline filled with color. Pink color represents female, while blue represent male mice. Values are expressed as Mean ± SEM, one-way ANOVA (Kruskal-Wallis test).

Stimulation (µA)	Male			
	Fiber Volley (mV)			
	Pre		Post	
	Sal (n=5)	Inh (n=6)	Sal (n=5)	Inh (n=6)
100	0.016 ± 0.040	0.013 ± 0.003	0.014 ± 0.007	0.013 ± 0.005
200	0.066 ± 0.011	0.082 ± 0.007	0.076 ± 0.020	0.085 ± 0.009
300	0.136 ± 0.028	0.172 ± 0.023	0.156 ± 0.027	0.192 ± 0.024
400	0.186 ± 0.041	0.217 ± 0.026	0.212 ± 0.034	0.265 ± 0.032
500	0.216 ± 0.047	0.228 ± 0.027	0.264 ± 0.045	0.293 ± 0.031
600	0.232 ± 0.053	0.242 ± 0.024	0.296 ± 0.053	0.310 ± 0.031
700	0.240 ± 0.059	0.253 ± 0.024	0.318 ± 0.060	0.322 ± 0.029
800	0.258 ± 0.059	0.257 ± 0.026	0.336 ± 0.064	0.330 ± 0.030
900	0.264 ± 0.059	0.262 ± 0.026	0.354 ± 0.067	0.345 ± 0.035
1000	0.270 ± 0.058	0.260 ± 0.027	0.366 ± 0.067	0.363 ± 0.043
	Slope (mV/ms)			
100	0.004 ± 0.002	0.002 ± 0.002	0.004 ± 0.002	0.002 ± 0.002
200	0.014 ± 0.002	0.020 ± 0.004	0.028 ± 0.009	0.050 ± 0.013
300	0.038 ± 0.006	0.037 ± 0.008	0.088 ± 0.022	0.095 ± 0.020
400	0.046 ± 0.007	0.047 ± 0.008	0.122 ± 0.032	0.132 ± 0.030
500	0.054 ± 0.010	0.052 ± 0.009	0.146 ± 0.039	0.155 ± 0.037
600	0.056 ± 0.009	0.057 ± 0.010	0.158 ± 0.039	0.178 ± 0.047
700	0.062 ± 0.009	0.060 ± 0.011	0.170 ± 0.039	0.198 ± 0.055
800	0.060 ± 0.009	0.060 ± 0.011	0.176 ± 0.040	0.210 ± 0.063
900	0.058 ± 0.007	0.062 ± 0.012	0.182 ± 0.036	0.218 ± 0.063
1000	0.060 ± 0.008	0.060 ± 0.012	0.186 ± 0.036	0.220 ± 0.065

	Female			
	Fiber Volley (mV)			
	Sal (n=6)	Inh (n=4)	Sal (n=6)	Inh (n=4)
100	0.025 ± 0.011	0.043 ± 0.017	0.028 ± 0.005	0.030 ± 0.009
200	0.068 ± 0.019	0.128 ± 0.068	0.080 ± 0.019	0.083 ± 0.018
300	0.117 ± 0.024	0.215 ± 0.104	0.150 ± 0.028	0.185 ± 0.027
400	0.142 ± 0.029	0.250 ± 0.096	0.173 ± 0.037	0.265 ± 0.035
500	0.197 ± 0.037	0.270 ± 0.085	0.227 ± 0.042	0.298 ± 0.032
600	0.225 ± 0.044	0.285 ± 0.075	0.250 ± 0.048	0.333 ± 0.040
700	0.237 ± 0.046	0.310 ± 0.078	0.268 ± 0.043	0.368 ± 0.057
800	0.245 ± 0.043	0.325 ± 0.079	0.307 ± 0.067	0.395 ± 0.070
900	0.252 ± 0.044	0.330 ± 0.087	0.273 ± 0.042	0.415 ± 0.081
1000	0.243 ± 0.040	0.338 ± 0.092	0.387 ± 0.146	0.428 ± 0.095
	Slope (mV/ms)			
100	0.002 ± 0.002	0.003 ± 0.003	0.001 ± 0.001	0.001 ± 0.001
200	0.030 ± 0.004	0.018 ± 0.005	0.037 ± 0.008	0.113 ± 0.023
300	0.060 ± 0.012	0.038 ± 0.009	0.090 ± 0.010	0.223 ± 0.021
400	0.082 ± 0.014	0.053 ± 0.011	0.135 ± 0.021	0.273 ± 0.022
500	0.092 ± 0.017	0.065 ± 0.015	0.162 ± 0.030	0.283 ± 0.020
600	0.098 ± 0.016	0.070 ± 0.016	0.178 ± 0.040	0.280 ± 0.025
700	0.100 ± 0.015	0.075 ± 0.018	0.192 ± 0.048	0.285 ± 0.022
800	0.102 ± 0.017	0.083 ± 0.020	0.197 ± 0.051	0.288 ± 0.027
900	0.107 ± 0.015	0.085 ± 0.022	0.207 ± 0.055	0.290 ± 0.022
1000	0.107 ± 0.018	0.090 ± 0.024	0.212 ± 0.056	0.288 ± 0.025

Suppl. Table S1: Synaptic strength is not affected by the chronic regimen used for PLD1 inhibition studies in the 12-month-old 3xTg-AD mice at the Schaffer collateral synapse following HFS-LTP. The slices were subjected to assessment with increasing stimulation from 100 to 1000 µA and the corresponding fiber volley (mV) and slope (mV/ms) are provided for each treatment group before (pre) and after (post) stimulation. "Pre" indicates the values before the stimulation, while "post" indicates the values after the stimulation protocol. Colored number indicate saline treated group and colored cells indicate inhibitor-treated group. Blue color represents males while pink color represents female mice. Values are expressed as mean ± standard error of mean and the number of animals per group are provided as "n", one-way ANOVA (Kruskal-Wallis test).

Time (ms)	Male			
	Saline (Pre)	Inhibitor (Pre)	Saline (Post)	Inhibitor (Post)
	(n=5)	(n=6)	(n=5)	(n=6)
200	1.208 ± 0.100	1.208 ± 0.029	1.109 ± 0.190	1.023 ± 0.017
100	1.347 ± 0.095	1.302 ± 0.141	1.026 ± 0.089	1.030 ± 0.029
50	1.415 ± 0.198	1.257 ± 0.104	1.086 ± 0.155	0.978 ± 0.079
25	1.481 ± 0.177	1.163 ± 0.087	1.101 ± 0.399	0.862 ± 0.101

Time (ms)	Female			
	Saline (Pre)	Inhibitor (Pre)	Saline (Post)	Inhibitor (Post)
	(n=6)	(n=4)	(n=6)	(n=4)
200	1.192 ± 0.137	1.475 ± 0.276	1.135 ± 0.104	0.983 ± 0.046
100	1.112 ± 0.077	1.150 ± 0.071	1.012 ± 0.049	1.050 ± 0.055
50	1.027 ± 0.119	1.328 ± 0.169	0.980 ± 0.050	1.060 ± 0.101
25	1.192 ± 0.226	1.143 ± 0.102	0.980 ± 0.066	0.983 ± 0.121

Suppl. Table S2: Paired pulse facilitation does not show differences between the treatment groups. The slices were subjected to pre- and post-HFS assessment of presynaptic vesicle release by estimating whether there is a change in the slope between the first pulse and the second pulse when the time

interval between the pulses is decreased from 200 ms to 100 ms to 50 ms to 25 ms. Colored number indicate saline treated group and colored cells indicate inhibitor-treated group. Blue color represents males while pink color represents female mice. Values are expressed as mean PPR ± standard error of mean and the number of animals per group are provided as "n". "Pre" indicates the values before the stimulation, while "post" indicates the values after the stimulation protocol, one-way ANOVA (Kruskal-Wallis test).

Male				
Stimulation (μA)	Fiber Volley (mV)			
	Pre		Post	
	Sal (n=4)	Inh (n=6)	Sal (n=4)	Inh (n=6)
100	0.010 ± 0.010	0.012 ± 0.002	0.015 ± 0.005	0.012 ± 0.005
200	0.070 ± 0.010	0.082 ± 0.012	0.040 ± 0.010	0.068 ± 0.015
300	0.165 ± 0.015	0.215 ± 0.028	0.145 ± 0.005	0.178 ± 0.024
400	0.230 ± 0.001	0.282 ± 0.035	0.220 ± 0.001	0.248 ± 0.033
500	0.295 ± 0.005	0.317 ± 0.040	0.270 ± 0.020	0.273 ± 0.033
600	0.325 ± 0.025	0.337 ± 0.041	0.305 ± 0.035	0.290 ± 0.033
700	0.345 ± 0.045	0.345 ± 0.041	0.320 ± 0.050	0.298 ± 0.031
800	0.365 ± 0.055	0.357 ± 0.039	0.345 ± 0.065	0.308 ± 0.032
900	0.385 ± 0.065	0.367 ± 0.041	0.350 ± 0.070	0.318 ± 0.031
1000	0.395 ± 0.085	0.373 ± 0.042	0.360 ± 0.070	0.328 ± 0.032
	Slope (mV/ms)			
100	0.048 ± 0.006	0.002 ± 0.002	0.008 ± 0.005	0.003 ± 0.002
200	0.093 ± 0.013	0.052 ± 0.016	0.040 ± 0.007	0.025 ± 0.011
300	0.153 ± 0.025	0.118 ± 0.043	0.225 ± 0.2658	0.062 ± 0.017
400	0.175 ± 0.025	0.158 ± 0.048	0.188 ± 0.0287	0.085 ± 0.017
500	0.208 ± 0.029	0.175 ± 0.044	0.245 ± 0.044	0.100 ± 0.016
600	0.250 ± 0.050	0.185 ± 0.044	0.303 ± 0.083	0.113 ± 0.017
700	0.273 ± 0.069	0.190 ± 0.044	0.328 ± 0.094	0.117 ± 0.016
800	0.283 ± 0.076	0.190 ± 0.041	0.340 ± 0.097	0.117 ± 0.015
900	0.288± 0.078	0.193 ± 0.042	0.353 ± 0.106	0.123 ± 0.018
1000	0.295 ± 0.085	0.193 ± 0.040	0.350 ± 0.110	0.125 ± 0.016

Female				
	Fiber Volley (mV)			
	Sal (n=4)	Inh (n=3)	Sal (n=4)	Inh (n=3)
100	0.018 ± 0.004	0.017 ± 0.003	0.015 ± 0.004	0.013 ± 0.003
200	0.058 ± 0.016	0.100 ± 0.017	0.033 ± 0.008	0.057 ± 0.017
300	0.162 ± 0.032	0.230 ± 0.061	0.120 ± 0.031	0.220 ± 0.071
400	0.248 ± 0.046	0.373 ± 0.133	0.213 ± 0.054	0.360 ± 0.132
500	0.313 ± 0.059	0.430 ± 0.162	0.273 ± 0.065	0.450 ± 0.207
600	0.352 ± 0.067	0.510 ± 0.222	0.318 ± 0.070	0.510 ± 0.262
700	0.380 ± 0.073	0.557 ± 0.250	0.345 ± 0.074	0.537 ± 0.279
800	0.392 ± 0.074	0.577 ± 0.255	0.368 ± 0.081	0.550 ± 0.292
900	0.405 ± 0.076	0.600 ± 0.270	0.383 ± 0.083	0.567 ± 0.309
1000	0.415 ± 0.075	0.620 ± 0.285	0.400 ± 0.083	0.573 ± 0.311
	Slope (mV/ms)			
100	0.053 ± 0.013	0.010 ± 0.010	0.015 ± 0.012	0.000 ± 0.000
200	0.100 ± 0.008	0.063 ± 0.029	0.083 ± 0.041	0.013 ± 0.003
300	0.198 ± 0.035	0.137 ± 0.056	0.168 ± 0.065	0.037 ± 0.009
400	0.285 ± 0.047	0.170 ± 0.053	0.218 ± 0.059	0.043 ± 0.012
500	0.325 ± 0.043	0.187 ± 0.045	0.255 ± 0.053	0.050 ± 0.010
600	0.368 ± 0.034	0.187 ± 0.034	0.280 ± 0.050	0.053 ± 0.012
700	0.395 ± 0.033	0.207 ± 0.024	0.295 ± 0.052	0.057 ± 0.009
800	0.420 ± 0.040	0.207 ± 0.019	0.305 ± 0.050	0.057 ± 0.009
900	0.435 ± 0.042	0.193 ± 0.022	0.308 ± 0.051	0.060 ±0.006
1000	0.445 ± 0.046	0.207 ± 0.013	0.323 ± 0.047	0.060 ± 0.006

Suppl. Table S3: Synaptic strength is not affected by the chronic regimen used for PLD1 inhibition studies in the 12-month-old 3xTg-AD mice at the Schaffer collateral synapse following LFS-LTD. The slices were subjected to assessment with increasing stimulation from 100 to 1000 μA and the corresponding fiber volley (mV) and slope (mV/ms) are provided for each treatment group before (pre) and after (post) stimulation. "Pre" indicates the values before the stimulation, while "post" indicates the values after the stimulation protocol. Colored number indicate saline treated group and colored cells indicate inhibitor-treated group. Blue color represents males while pink color represents female mice. Values are expressed as mean ± standard error of mean and the number of animals per group are provided as "n", one-way ANOVA (Kruskal-Wallis test).

Time (ms)	Male			
	Saline (Pre)	Inhibitor (Pre)	Saline (Post)	Inhibitor (Post)
	(n=4)	(n=6)	(n=4)	(n=6)
200	0.835 ± 0.104	0.922 ± 0.044	0.893 ± 0.043	0.850 ± 0.031
100	0.910 ± 0.052	0.838 ± 0.027	0.895 ± 0.034	0.792 ± 0.034
50	0.805 ± 0.027	0.805 ± 0.068	0.845 ± 0.056	0.855 ± 0.105
25	0.655 ± 0.111	0.638 ± 0.048	0.673 ± 0.101	0.610 ± 0.037

Time (ms)	Female			
	Saline (Pre)	Inhibitor (Pre)	Saline (Post)	Inhibitor (Post)
	(n=4)	(n=3)	(n=4)	(n=3)
200	1.023 ± 0.171	1.100 ± 0.107	1.133 ± 0.226	0.943 ± 0.096
100	1.068 ± 0.128	1.087 ± 0.127	0.925 ± 0.077	1.097 ± 0.035
50	0.993 ± 0.087	0.970 ± 0.184	0.845 ± 0.047	1.007 ± 0.153
25	0.908 ± 0.114	0.893 ± 0.166	0.698 ± 0.031	0.963 ± 0.209

Suppl. Table S4: Paired pulse depression does not show differences between the treatment groups. The slices were subjected to pre- and post-LFS assessment of presynaptic vesicle release by estimating whether there is a change in the slope between the first pulse and the second pulse when the time interval between the pulses is decreased from 200 ms to 100 ms to 50 ms to 25 ms. "Pre" indicates the values before the stimulation, while "post" indicates the values after the stimulation protocol. Colored number indicate saline treated group and colored cells indicate inhibitor-treated group. Blue color represents males while pink color represents female mice. Values are expressed as mean PPR ± standard error of mean and the number of animals per group are provided as "n", one-way ANOVA (Kruskal-Wallis test).
